# Ciraparantag reverses the anticoagulant activity of apixaban and rivaroxaban in healthy elderly subjects

**DOI:** 10.1093/eurheartj/ehab637

**Published:** 2021-09-17

**Authors:** Jack Ansell, Sasha Bakhru, Bryan E Laulicht, Gregory Tracey, Stephen Villano, Daniel Freedman

**Affiliations:** Hofstra Northwell School of Medicine, 500 Hofstra Blvd., Hempstead, NY 11549, USA; Perosphere Technologies Inc., 108 Mill Plain Rd., Danbury, CT 06811, USA; Landsdowne Laboratories Inc., 1073 N. Benson Rd., Fairfield, CT 06824, USA; Frontage Clinical Services, Inc., 200 Meadowlands Pkwy, Secaucus, NJ 07094, USA; AMAG Pharmaceuticals, Inc., 1100 Winter St., Waltham, MA 02450, USA; AMAG Pharmaceuticals, Inc., 1100 Winter St., Waltham, MA 02450, USA

**Keywords:** Anticoagulant, Antidote, Apixaban, Ciraparantag, Rivaroxaban

## Abstract

**Aims:**

Ciraparantag is a reversal agent for anticoagulants including direct oral anticoagulants. The aim was to evaluate the efficacy and safety of ciraparantag to reverse anticoagulation induced by apixaban or rivaroxaban in healthy elderly adults.

**Methods and results:**

Two randomized, placebo-controlled, dose-ranging trials conducted in healthy subjects aged 50–75 years. Subjects received apixaban (Study 1) 10 mg orally twice daily for 3.5 days or rivaroxaban (Study 2) 20 mg orally once daily for 3 days. At steady-state anticoagulation subjects were randomized 3:1 to a single intravenous dose of ciraparantag (Study 1: 30, 60, or 120 mg; Study 2: 30, 60, 120, or 180 mg) or placebo. Efficacy was based on correction of the whole blood clotting time (WBCT) at multiple timepoints over 24 h. Subjects and technicians performing WBCT testing were blinded to treatment. Complete reversal of WBCT within 1 h post-dose and sustained through 5 h (apixaban) or 6 h (rivaroxaban) was dose related and observed with apixaban in 67%, 100%, 100%, and 17% of subjects receiving ciraparantag 30 mg, 60 mg, 120 mg, or placebo, respectively; and with rivaroxaban in 58%, 75%, 67%, 100%, and 13% of subjects receiving ciraparantag 30 mg, 60 mg, 120 mg, 180 mg, or placebo, respectively. Adverse events related to ciraparantag were mild, transient hot flashes or flushing.

**Conclusions:**

Ciraparantag provides a dose-related reversal of anticoagulation induced by steady-state dosing of apixaban or rivaroxaban. Sustained reversal was achieved with 60 mg ciraparantag for apixaban and 180 mg ciraparantag for rivaroxaban. All doses of ciraparantag were well tolerated.

## Graphical Abstract

**Graphical Abstract:**
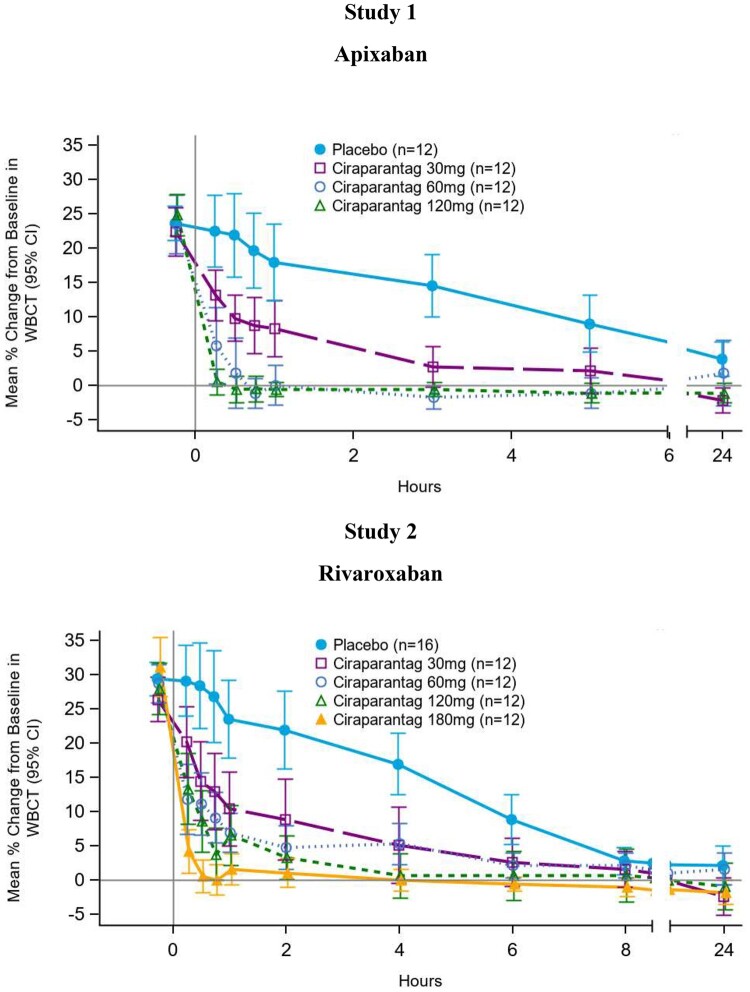
Percent change from baseline in whole blood clotting time over time. The first displayed timepoint represents the time of peak anticoagulant effect, measured ∼15 min prior to study drug infusion. Time 0 represents the end of study drug infusion. CI, confidence interval; WBCT, whole blood clotting time.

**See the editorial comment for this article ‘Ciraparantag as a potential universal anticoagulant reversal agent’, by Noel C. Chan and Jeffrey I.Weitz, https://doi.org/10.1093/eurheartj/ehab706.**

## Introduction

Direct oral anticoagulants (DOACs) are now recognized as the first-line treatments for stroke prevention in atrial fibrillation and for the acute treatment and long-term prevention of recurrent venous thromboembolism.^1–3^ The first DOAC to be approved in the USA was dabigatran etexilate in 2009, followed by the factor Xa (FXa) inhibitors (apixaban, betrixaban, edoxaban, and rivaroxaban). Although DOACs have become the dominant means of chronic anticoagulation over warfarin,^1^,^4^ until recently specific reversal agents were not available.^5^ This situation is now partially remedied with a humanized murine monoclonal antibody Fab fragment (Praxbind^®^, Boehringer Ingelheim) targeted to bind to and reverse dabigatran.^6^ A second agent recently approved, andexanet alfa (Andexxa^®^, Alexion Pharmaceuticals, Inc.), is a modified, enzymatically inactive recombinant FXa functioning as a decoy Xa targeted to bind to and reverse the oral FXa inhibitors as well as enoxaparin,^7^ but it has some challenges to its widespread use.^8^ Hence, there remains an unmet need for agents that can rapidly reverse the effects of DOACs in emergency settings.

Ciraparantag (COVIS Pharma) is an investigational compound being developed as an anticoagulant reversal agent. Ciraparantag is a small synthetic water-soluble molecule with broad activity, reversing both the oral direct FXa inhibitors and the parenteral indirect FXa and FIIa inhibitor enoxaparin.^9–11^ Ciraparantag directly binds to DOACs and to enoxaparin through non-covalent hydrogen bonds and charge–charge interactions, removing these drugs from their intended target site and reversing their anticoagulant effects.^9^

Two Phase 2 clinical trials were conducted to evaluate the efficacy and safety of ciraparantag in healthy elderly subjects who were anticoagulated to steady state with apixaban (Study 1) and rivaroxaban (Study 2) using the manual whole blood clotting time (WBCT) as a measure of coagulation activity. These dose-ranging studies were intended to identify a dose of ciraparantag that fully reverses apixaban and rivaroxaban at steady-state concentrations in healthy subjects.

## Methods

The two studies were randomized, single-blind, placebo-controlled, Phase 2 clinical trials (www.clinicaltrials.gov # NCT03288454 and # NCT03172910). The objectives of the studies were the following: to evaluate the safety and tolerability of escalating intravenous (IV) doses of ciraparantag; and to evaluate the pharmacodynamic effects of these ciraparantag doses administered 3 h after the Day 3 dose of steady-state apixaban (Study 1) or rivaroxaban (Study 2) as measured by serial manual WBCT. Pharmacokinetic assessments of ciraparantag, apixaban (Study 1), and rivaroxaban (Study 2), and their major metabolites were performed but are not presented in this report.

Healthy non-smoking subjects, aged 50–75 years (inclusive) were eligible to participate in the studies. Subjects were excluded from the studies if they had any significant clinical medical history including family or personal history of clotting abnormality or excessive bleeding, thrombotic or vascular disease, personal history of major or minor bleeding episodes within 3–6 months prior to screening, blood product or anticoagulant use within the prior 3 months, non-steroidal anti-inflammatory drug use within the prior 2 weeks, use of chronic medication or history of drug or alcohol dependence, were positive for human immunodeficiency virus or hepatitis B or C, were pregnant, or participated in any previous ciraparantag study. After informed consent and screening, subjects were confined to a clinical study unit for 5 days (Study 1) or 6 days (Study 2) starting on the day prior to anticoagulant dosing. Enrolled subjects were treated with apixaban (10 mg orally, twice daily for 3.5 days; Eliquis^®^; Bristol-Myers Squibb) or rivaroxaban (20 mg orally, once daily for 3 days; Xarelto^®^; Janssen Pharmaceuticals, Inc.) to steady-state anticoagulation. In Study 1, steady-state anticoagulation was defined as WBCT ≥20% above baseline 2.75 h after last apixaban dose. In Study 2, steady-state anticoagulation was defined as WBCT ≥25% above baseline 3.75 h after last rivaroxaban dose. These levels represent anticoagulant drug levels of ∼150 ng/mL or more. Patients who did not reach criteria for anticoagulation following their apixaban or rivaroxaban treatment were discontinued from the trial and replaced. Key design elements of the two studies are shown in *Table 1*.

**Table 1:** Summary of key design elements

	Study 1	Study 2
	Apixaban	Rivaroxaban
Anticoagulant regimen	Apixaban 10 mg orally, twice daily for 3.5 days	Rivaroxaban 20 mg orally, once daily for 3 days
Criteria for sufficient anticoagulation for randomization	2.75 h after last apixaban dose, WBCT ≥20% above baseline	3.75 h after last rivaroxaban dose, WBCT ≥25% above baseline
Timing of ciraparantag/placebo after last anticoagulant dose	3 h	4 h
Ciraparantag doses (mg)^a^	30, 60, 120	30, 60, 120, 180
WBCT timepoints after ciraparantag/placebo (h)	0.25, 0.5, 0.75, 1, 3, 5, 24	0.25, 0.5, 0.75, 1, 2, 4, 6, 8, 24
‘Responder’ defined as WBCT ≤10% above baseline within 1 h and sustained after 1 h through X h	X = at least 5 h	X = at least 6 h

WBCT, whole blood clotting time.

a Active study drug (ciraparantag) doses are expressed as active drug moiety.

Subjects who reached steady-state anticoagulation were randomized, in blinded fashion, to receive either ciraparantag or placebo in a 3:1 ratio (*n* = 16 per dose cohort). In Study 1 (apixaban), three dosing cohorts of ciraparantag were studied (30, 60, and 120 mg), each initially intended to enroll at least 16 subjects. In Study 2 (rivaroxaban), four dosing cohorts of ciraparantag were studied (30, 60, 120, and 180 mg), each initially intended to enroll at least 16 subjects. Study drug (ciraparantag or placebo) was administered as a single IV infusion over 10 min at either 3 h (Study 1, apixaban) or 4 h (Study 2, rivaroxaban) after the last dose of the anticoagulant.

In both studies, serial testing of the manual WBCT was used as the primary measure of coagulation activity and the efficacy of ciraparantag. In earlier studies, it was found that using standard assays (e.g. prothrombin time, activated partial thromboplastin time, anti-Xa assay, TEG-R^®^, and Hemochron Signature^®^), all of which required plasma or whole blood with activators, resulted in variable or no changes in degree of anticoagulation. This was due to the binding of ciraparantag in the test tube with reagents used to anticoagulate blood in order to derive plasma (or to activate coagulation). Thus, ciraparantag cannot be studied in plasma from blood collected with sodium citrate, oxalate, or heparin. The molar excess of these anions overwhelms and disrupts the ciraparantag anticoagulant complex, thereby freeing the anticoagulant in the plasma and making plasma-based assays non-representative of physiological conditions. Kaolin and celite activators also adsorb ciraparantag, significantly reducing the active concentration of soluble ciraparantag in a blood sample and making kaolin and celite-based assays insensitive to quantitating reversal by ciraparantag. Thus, the manual WBCT, in which no reagent is required, was used as the biomarker to measure anticoagulation and its reversal. Subjects and technicians performing the WBCT testing were blinded to treatment. WBCT measures were performed in triplicate (simultaneous testing by three different evaluators) at three separate timepoints (i.e. baseline, pre-dose, and 1 h post-dose).

The primary efficacy endpoint with each study was the percent change in coagulant activity as measured by WBCT. Efficacy analyses, detailed below, focused on reversal of anticoagulation. ‘Complete reversal’ of anticoagulation was defined as a return of WBCT to ≤10% above baseline at any time point within 1 h of study drug administration. This cut-off was based on earlier studies indicating it represented drug levels of ∼75 ng/mL or less. ‘Complete and sustained reversal’ was defined as a return of mean manual WBCT to ≤10% above baseline during all time points between 1 and 5 h (Study 1) or 6 h (Study 2) after administration of study drug.

Safety evaluations included a continuous assessment of adverse events (AEs), and intermittent assessments of vital signs, electrocardiograms (ECGs), and standard laboratory testing (chemistry, haematology, and urinalysis). On the final day in the study unit, a physical exam and safety laboratory samples were obtained prior to discharge from the study unit. Follow-up contact (phone call) was made between Days 7 and 10 to assess any AEs. In both studies, doses of anticoagulant (apixaban or rivaroxaban) were administered in an open-label manner. Study subjects and site personnel performing coagulation testing and reporting WBCT measurements were blinded to treatment (ciraparantag or placebo). The Investigator and the Sponsor were unblinded.

### Study oversight

Each study was performed at a single site in the USA (Frontage Clinical Services, Inc., Secaucus, NJ, USA) and both were sponsored by Perosphere Pharmaceuticals Inc. (now a wholly owned subsidiary of AMAG Pharmaceuticals Inc.). Both trials were conducted in accordance with Good Clinical Practice guidelines and the provisions of the Declaration of Helsinki. All subjects provided written informed consent prior to participation in the studies. Qualified representatives monitored the trials according to the predetermined monitoring plan to verify protocol adherence and accuracy and completeness of data. Both protocols were approved by the independent Investigational Review Board, IntegReview IRB (Austin, TX, USA). Data collection and entry were performed by Frontage Clinical Services, Inc. where the study was conducted. Data analysis was performed by the authors and Frontage Clinical Services, Inc. The authors had full access to the data and analyses for compilation of this report. Manuscript drafts were prepared by the authors and all authors made the decision to submit the manuscript for publication and vouch for the accuracy and completeness of the data reported.

### Statistical analysis

A formal sample size calculation was not performed for this study and 16 subjects enrolled in each cohort (3:1; active: placebo) was considered an adequate number to achieve study objectives. The pharmacodynamic analysis set includes all subjects who receive at least one administration of the investigational product, and provides at least one on-treatment WBCT measurement without protocol deviations with potential to affect these measurements. The safety analysis set includes all subjects who receive at least one administration of the investigational product. This set will be used for all safety, demographic, subject, and disposition summaries.

All evaluable data from subjects in the analysis sets were to be included in the analyses. No adjustment or imputation was utilized for missing values or for subjects who withdrew prior to completing the study, neither were analyses to be restricted to subjects with complete data. For WBCT, baseline values were to be defined as the pre-apixaban or rivaroxaban sample taken on the morning of Day 3. For the timepoints at which WBCT was measured in triplicate, the median values were derived and used for analysis in this study. Statistically significant complete reversal, relative to mean placebo WBCT, was defined as *P*-value <0.05 (one-tailed) from the comparison of means between treatment and placebo at 15 min, 30 min, 45 min, or 1 h post-study drug administration as measured by one-way analysis of variance at study completion.

## Results

### Subjects

**Figure 1:**
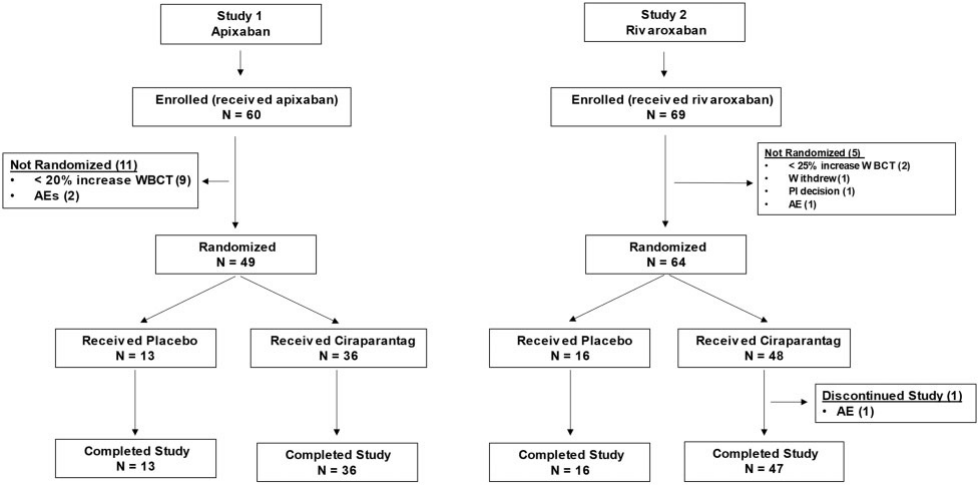
Subject disposition. AE, adverse event; WBCT, whole blood clotting time.

In Study 1, 60 subjects were enrolled and received at least one dose of apixaban. Of these, 11 subjects were not randomized to study drug (9 did not achieve the defined level of anticoagulation, and 2 had AEs during the apixaban administration period causing discontinuation). Forty-nine subjects were randomized (36 ciraparantag, 13 placebo); all completed the trial as planned (*Figure 1*).

In Study 2, 69 subjects were enrolled and received at least one dose of rivaroxaban. Of these, five were not randomized (two did not achieve the defined level of anticoagulation, one discontinued per investigator discretion, one withdrew consent, and one had an AE). Therefore, 64 subjects were randomized (48 ciraparantag, 16 placebo); all but one subject (who had an unrelated AE) completed the trial as planned (*Figure 1*).

There were no important differences in demographic or clinical characteristics between any of the study groups as shown in *Table 2*.

**Table 2:** Subject demographics

Characteristics	Study 1		Study 2	
	Apixaban		Rivaroxaban	
	Ciraparantag (*n* = 36)	Placebo (*n* = 13)	Ciraparantag (*n* = 48)	Placebo (*n* = 16)
Age (years), mean (SD)	54.7 (4.1)	57.2 (6.9)	55.9 (5.3)	54.3 (3.9)
Age range (years)	50–64	50–73	50–73	50–65
Male sex, *n* (%)	28 (77.8)	10 (76.9)	41 (85.4)	14 (87.5)
Weight (kg), mean (SD)	77.9 (11.5)	82.0 (16.8)	83.6 (13.9)	78.7 (14.3)
BMI (kg/m^2^), mean (SD)	26.4 (2.8)	27.4 (3.7)	27.7 (2.9)	26.0 (3.5)
GFR (mL/min), mean (SD)	85.1 (12.6)	81.5 (13.9)	84.0 (10.68)	85.6 (13.3)
Ethnicity, *n* (%)^a^				
White	18 (50.0)	8 (61.5)	24 (50.0)	8 (50)
Black or African American	16 (44.4)	5 (38.5)	22 (45.8)	8 (50)
Hispanic or Latino	9 (25.0)	5 (38.5)	11 (22.9)	5 (31.3)
Asian	2 (5.6)	5 (38.5)	2 (4.2)	0

BMI, body mass index; GFR, glomerular filtration rate; SD, standard deviation.

a Ethnicity numbers greater than total ‘*n*’ because of overlap in subjects.

### Pharmacodynamics

Ciraparantag produced a rapid and dose-related reversal of anticoagulation induced by apixaban and rivaroxaban compared with placebo as measured by WBCT (*Graphical abstract*). In both studies, analysis of least squares mean WBCT values showed statistically significant differences between each ciraparantag group and placebo at each timepoint through 1 h after dosing.

**Figure 2:**
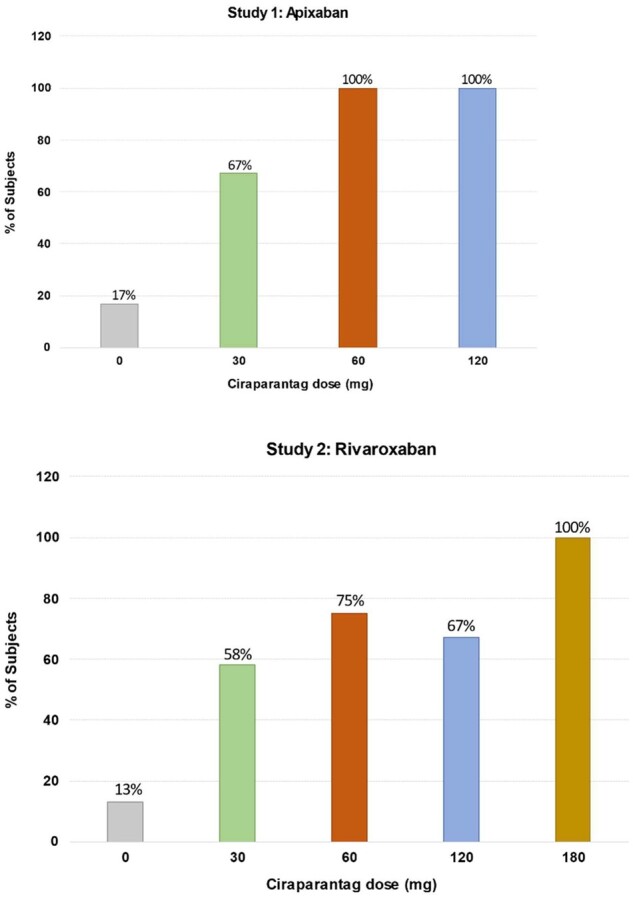
Correction of whole blood clotting time vs. dose. Proportion of subjects with complete and sustained reversal of steady-state anticoagulation induced by apixaban (Study 1) or rivaroxaban (Study 2) based on manual whole blood clotting time vs. dose. In this analysis, complete and sustained reversal is a whole blood clotting time ≤10% above baseline within 1 h after ciraparantag/placebo dose and sustained through at least 5 or 6 h for apixaban or rivaroxaban, respectively.

Complete reversal of WBCT to ≤10% of baseline within 1 h post-dose and sustained through 5 h (apixaban) or 6 h (rivaroxaban) is illustrated in *Figure 2*. The effect was dose related and observed with apixaban (Study 1) in 67%, 100%, 100%, and 17% of subjects receiving ciraparantag 30 mg, 60 mg, 120 mg, or placebo, respectively; and with rivaroxaban (Study 2) in 58%, 75%, 67%, 100%, and 13% of subjects receiving ciraparantag 30 mg, 60 mg, 120 mg, 180 mg, or placebo, respectively.

**Figure 3:**
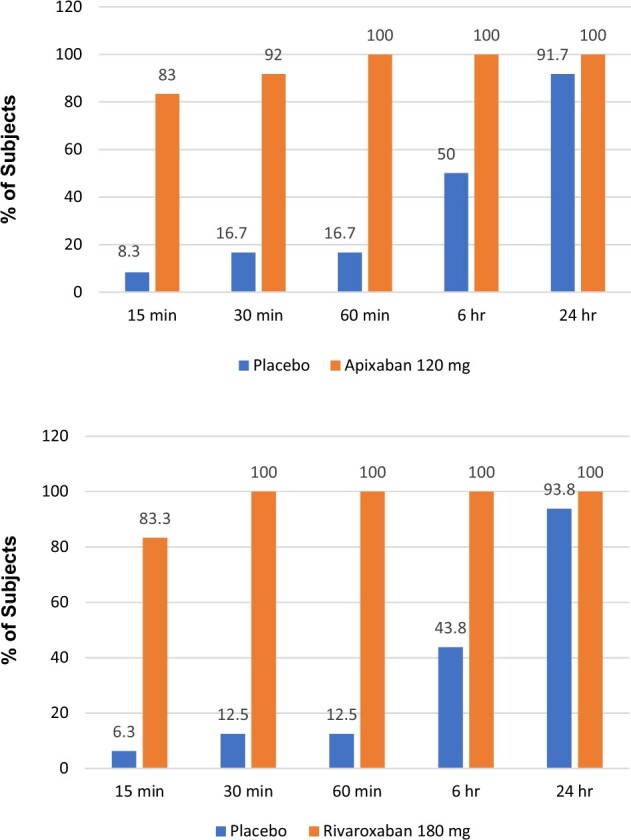
Correction of whole blood clotting time vs. time. Proportion of subjects with complete reversal of steady-state anticoagulation induced by apixaban (Study 1) or rivaroxaban (Study 2) based on manual whole blood clotting time vs. time. In this analysis, complete reversal is defined as a whole blood clotting time ≤10% above baseline at any time point within 1 h of ciraparantag/placebo dose. The dose of ciraparantag for apixaban was 120 mg and for rivaroxaban was 180 mg.

At the highest doses of ciraparantag studied (120 mg for apixaban, 180 mg for rivaroxaban), complete reversal of anticoagulation within the first hour to ≤10% above baseline occurred in 83%, 92%, and 100% of apixaban subjects at 15, 30, and 60 min, respectively. For rivaroxaban, complete reversal occurred in 83%, 100%, and 100% of subjects at 15, 30, and 60 min, respectively (*Figure 3*).

### Safety and tolerability

Ciraparantag was well tolerated across the range of doses evaluated. Treatment-emergent AEs are summarized in *Table 3*. Across both trials, all treatment-related AEs were mild except for one event of supraventricular tachycardia in a ciraparantag subject (180 mg dose) in the rivaroxaban trial that was considered not related to study drug (the subject had a history of supraventricular tachycardia which had not been disclosed during trial screening). The AE was severe and was the only serious AE.

**Table 3:** Treatment-emergent adverse events

	Study 1		Study 2	
	Apixaban		Rivaroxaban	
Subjects with	Ciraparantag (*n* = 36)	Placebo (*n* = 13)	Ciraparantag (*n* = 48)	Placebo (*n* = 16)
TEAEs	13 (36.1%)	0	20 (41.7%)	2 (12.5%)
TEAEs in >1 subject				
Hot flush	8 (22.2%)	0	9 (18.8%)	0
Feeling hot	3 (8.3%)	0	2 (4.2%)	0
Feeling cold	1 (2.8%)	0	4 (8.3%)	0
Paraesthesia	0	0	3 (6.3%)	0
Flushing	0	0	2 (4.2%)	0
Dizziness	0	0	2 (4.2%)	0
Dysgeusia	0	0	2 (4.2%)	0

TEAE, treatment-emergent adverse event.

The most common AEs were mild, transient sensations of warmth (reported as hot flashes, feeling hot, or flushing), which were dose related. Across both trials, any such event occurred in 4%, 21%, 42%, and 67% of those in the 30, 60, 120, and 180 mg groups, respectively. All of these events were mild; they occurred within minutes after the start of study drug infusion and resolved spontaneously (83% resolved within <1 h). There were no clinically significant findings in laboratory test results, vital signs, or ECG parameters.

## Discussion

In these two randomized, single-blind, placebo-controlled Phase 2 clinical trials, ciraparantag was shown to be safe and well tolerated at all doses studied as it had been in previous trials. The most common side effect, vascular flushing, was similar to findings in a Phase 1–2 trial of reversal of edoxaban^10^ and enoxaparin^11^ and was not a dose limiting side effect. Most importantly, ciraparantag was shown to reverse the anticoagulant effect induced by steady-state dosing of apixaban and rivaroxaban in healthy subjects and maintain reversal over a minimum of 5 and 6 h, respectively (the last measurement before the 24 h measurement). The reversal effect was dose dependent, with higher doses required for full reversal of rivaroxaban than for apixaban.

As shown in the initial preclinical studies,^9^ plasma-based assays are unsuitable for measuring the effect of ciraparantag on anticoagulation reversal. Thus, the manual WBCT was used as the biomarker to measure anticoagulation and its reversal. Manual WBCT is a global measure of clotting that reflects both impaired coagulation induced by various anticoagulants including DOACs and the return to normal coagulation induced by a DOAC reversal agent such as ciraparantag. Manual WBCT in the hands of trained technicians shows high precision and reproducibility. In both studies, there was good agreement among the triplicate manual WBCT measurements (coefficient of variance % apixaban study: placebo 2.1–2.5, 30 mg dose 2.2–2.9, 60 mg dose 2.1–4.8, 120 mg dose 2.5–2.6; similar results were obtained for rivaroxaban study). All inter-observer coefficients of variance values were <5%.

The currently available agents for reversal of DOACs have several shortcomings. Idarucizumab is a specific reversal agent targeted only to dabigatran.^5^,^6^ It has shown good efficacy and safety, but as a biologic, it is expensive, and it has limited use in that dabigatran occupies a shrinking share of the anticoagulant market. Andexanet alfa not only has efficacy in reversing the oral FXa inhibitors and enoxaparin^5^,^7^ but also has drawbacks including high cost, limited duration of effect requiring a continuous infusion, possible evidence of a procoagulant effect,^12^ extensive preparation time required, and limited availability. Prothrombin complex concentrate, commonly used to reverse vitamin K antagonists, has shown effectiveness in reversing the anticoagulant effect of DOACs as well.^13^ These concentrates, however, are also prothrombotic and expensive and require some time for preparation. Alternatively, ciraparantag addresses many of these drawbacks as a small molecule with a long duration of effect after a brief IV infusion, with no evidence of a prothrombotic signal and with a stable shelf-life and an agent that is easily and rapidly prepared for injection.

## Conclusion

In these two Phase 2 trials, ciraparantag provided a dose-related reversal of anticoagulation induced by steady-state dosing of apixaban or rivaroxaban. Doses of 60 and 120 mg provided a rapid and sustained reversal in all subjects treated with apixaban, and 180 mg provided a rapid and sustained reversal for all subjects treated with rivaroxaban. All doses of ciraparantag were well tolerated by trial participants.

## Notes

All work was performed at Frontage Clinical Services, Inc., Secaucus, NJ, USA.
